# 3D Features Fusion for Automated Segmentation of Fluid Regions in CSCR Patients: An OCT-based Photodynamic Therapy Response Analysis

**DOI:** 10.1007/s10278-024-01190-y

**Published:** 2024-07-29

**Authors:** Elena Goyanes, Joaquim de Moura, José I. Fernández-Vigo, Julián García-Feijóo, Jorge Novo, Marcos Ortega

**Affiliations:** 1 VARPA Research Group, Instituto de Investigación Biomédica de A Coruña (INIBIC), Universidade da Coruña, A Coruña, Spain; 2 Retina Unit, Ophthalmology Department, Hospital Clínico San Carlos, IdISSC, Madrid, Spain

**Keywords:** CAD system, Central serous chorioretinopathy, Photodynamic Therapy, OCT, 3D Segmentation, Deep Learning

## Abstract

Central Serous Chorioretinopathy (CSCR) is a significant cause of vision impairment worldwide, with Photodynamic Therapy (PDT) emerging as a promising treatment strategy. The capability to precisely segment fluid regions in Optical Coherence Tomography (OCT) scans and predict the response to PDT treatment can substantially augment patient outcomes. This paper introduces a novel deep learning (DL) methodology for automated 3D segmentation of fluid regions in OCT scans, followed by a subsequent PDT response analysis for CSCR patients. Our approach utilizes the rich 3D contextual information from OCT scans to train a model that accurately delineates fluid regions. This model not only substantially reduces the time and effort required for segmentation but also offers a standardized technique, fostering further large-scale research studies. Additionally, by incorporating pre- and post-treatment OCT scans, our model is capable of predicting PDT response, hence enabling the formulation of personalized treatment strategies and optimized patient management. To validate our approach, we employed a robust dataset comprising 2,769 OCT scans (124 3D volumes), and the results obtained were significantly satisfactory, outperforming the current state-of-the-art methods. This research signifies an important milestone in the integration of DL advancements with practical clinical applications, propelling us a step closer towards improved management of CSCR. Furthermore, the methodologies and systems developed can be adapted and extrapolated to tackle similar challenges in the diagnosis and treatment of other retinal pathologies, favoring more comprehensive and personalized patient care.

## Introduction

Central Serous Chorioretinopathy (CSCR) is a retinal disorder prevalent in the adult population, defined by fluid accumulation in the subretinal space, originated from the choroid layer of the eye [1–3]. This build-up can lead to potential decreases in visual acuity (VA), causing significant discomfort and disruption to the daily lives of affected individuals. Therefore, the need for accurate diagnosis and effective monitoring of CSCR is of paramount importance in mitigating visual impairment and improving patient outcomes. In this context, the development of an efficient, precise, and automated system capable of segmenting fluid regions in ocular scans represents a crucial breakthrough for diagnosing and managing CSCR.

Over the past years, Photodynamic Therapy (PDT) has emerged as a treatment of choice for CSCR due to its proven safety and efficacy [4–6]. PDT operates by selectively activating a photosensitizer, causing the creation of reactive oxygen species that target and destroy abnormal choroidal vasculature. This approach is highly compatible with the pathophysiology of CSCR, aiding in effective subretinal fluid (SRF) reduction and encouraging the restoration of retinal function.

The adoption of PDT is increasingly widespread, backed by a solid body of clinical evidence attesting to its effectiveness and safety. Scientific studies reveal a significant reduction in SRF following PDT administration, with associated improvements in VA [4]. However, the effectiveness of PDT is not uniformly seen across all patients, underscoring the need for an individualized treatment approach. Here, the segmentation of fluid regions in retinal images plays a crucial role in guiding the treatment plans.

Parallel to advancements in treatment options, Optical Coherence Tomography (OCT), a non-invasive imaging technique, has shown tremendous value for diagnosing CSCR [7–9]. OCT generates high-resolution images that enable the identification and quantification of SRF, a key indicator of CSCR. However, the evaluation of fluid regions in OCT scans currently relies heavily on manual intervention. This process is not only resource-intensive and time-consuming but also susceptible to variability due to its dependence on the expertise of the individual conducting the segmentation.

This study aims to circumvent these limitations by proposing an automated system for 3D fluid region segmentation in OCT scans of CSCR patients, leveraging the power of deep learning (DL). We envision this system drastically reducing segmentation time and effort, substantially improving efficiency, and enabling the analysis of large datasets. The system is also expected to produce standardized and consistent results, minimizing potential errors and variability. Consequently, it would significantly contribute to the accuracy in diagnosing CSCR, monitoring its progression, and assessing responses to treatments like PDT [10, 11].

Our study distinguishes itself through a keen focus on harnessing the volumetric, 3D information inherent in OCT scans. OCT scans capture an extensive range of data, providing a 3D perspective of the retina that is essential for a comprehensive understanding of the disease state. We hypothesize that incorporating this 3D context into our DL model will facilitate the extraction of nuanced features and comprehensive characterization of the fluid regions. Consequently, the derived insights may shed light on the evolution of these regions over time, thus offering an invaluable perspective on the progression of the disease and the effectiveness of treatments.

In addition to automating the segmentation process, this study extends its scope to predict the response of chronic CSCR patients to PDT based on the analysis of pre-treatment OCT scans. We endeavor to unearth patterns and correlations that may act as dependable indicators of treatment efficacy. The predictive power of this system has the potential to significantly enhance patient care. By foreseeing treatment responses, clinicians can make informed modifications to treatment plans, thereby optimizing patient outcomes. This proactive approach aids in personalizing therapeutic strategies and enables the efficient allocation of medical resources.

Furthermore, this system could prove to be an indispensable resource for researchers. It could facilitate large-scale, systematic studies into the impact of PDT on chronic CSCR, thus enhancing our comprehension of the disease and its treatments. Such investigations, otherwise hindered due to the extensive manual efforts required in data analysis, could potentially lead to the discovery of novel treatment methodologies and improved management strategies.

### Related works

In this subsection, we explore the existing body of literature pertinent to the two primary objectives of this work: fluid segmentation in CSCR and PDT response analysis. This overview will shed light on the methodologies and techniques previously employed in these areas, their successes and limitations, thereby establishing the context and necessity for the approach proposed in this study.

#### CSCR fluid segmentation

The automation of CSCR diagnosis using Computer-Aided Diagnosis (CAD) systems can notably enhance efficiency and accuracy, minimizing the errors that arise from subjective expert evaluations. A number of studies have delved into the use of CAD for CSCR diagnosis. For instance, the work by Chen et al. [12] proposed an attention-gated network DL model for the automatic detection of CSCR leakage points in fundus fluorescein angiography, showcasing the potential of DL models in this realm. Similarly, the research by Xu et al. [13] designed a DL-based framework for screening SRF from fundus images, employing a cascading approach with two Convolutional Neural Network (CNN) models. Yoo et al. [14] leveraged a CNN architecture for efficient SRF area segmentation in fundus photography, further validating the utility of DL in CSCR characterization.

In parallel, OCT has garnered attention for diagnosing retinal diseases, primarily due to its prowess in pathological fluid region detection. By harnessing high-resolution cross-sectional OCT images and automatic segmentation techniques, researchers have made substantial progress in analyzing various retinal diseases, including diabetic macular edema, glaucoma, and age-related macular degeneration [15–19].

Several recent studies have utilized OCT to automate CSCR analysis. Gao et al. [20] applied an area-constraint fully-convolutional network for the automatic segmentation of CSCR regions in OCT images, yielding results on par with manual segmentation, following independent layer segmentation as well as quantitative and qualitative evaluations. Rao et al. [21] improved the segmentation of regions using DL-based architectures coupled with a data pre-processing stage. De Moura et al. [22] proposed an end-to-end methodology employing a fully convolutional architecture to identify and segment intraretinal fluid regions associated with CSCR in OCT scans.

These studies bear witness to the strides made in automating the analysis of CSCR using OCT imaging. However, a closer examination reveals that the full potential of OCT scans, particularly their 3D volumetric information, remains underexplored in the context of fluid segmentation in CSCR. Furthermore, while these methods have proven effective, their efficiency and accuracy can still be improved. These uncharted territories provide the impetus for the current study, prompting us to propose a comprehensive DL-based approach for 3D fluid segmentation in CSCR.

#### Response to PDT

In clinical practice, the response to PDT varies considerably, and the prediction of which patients will experience a favorable treatment response with complete resorption of SRF remains a challenging task. The resolution of SRF with half-fluence PDT has been reported to range from 67% to 97% across different series [4, 6, 23, 24]. Several clinical factors, such as advanced age, low baseline VA or the extent of retinal pigment epithelium (RPE) damage, have been associated with a less favorable response to PDT [25]. However, there are few studies that quantify the predictive value of these characteristics. Furthermore, the specific baseline anatomical features observable in OCT scans that might determine a patient’s response to PDT remain largely unexplored.

Within the realm of ophthalmology, the applications of artificial intelligence (AI) have primarily been concentrated on improving image analysis and predicting clinical outcomes [26–28]. Despite the demonstrated success of DL in accurately identifying CSCR using fundus images and distinguishing between its acute and chronic forms through imaging analysis [29, 30], studies investigating the potential utility of AI in the analysis of CSCR using OCT have been limited. Recently, an AI-based study conducted by Xu et al. [31] exhibited that their DL and machine learning-based algorithms can predict VA and post-therapeutic OCT images in patients with CSCR. FernÃ¡ndez-Vigo et al. [32] in a recent publication presented a study that focused on predicting the response to PDT in CSCR patients by leveraging DL with spectral-domain optical coherence tomography (SD-OCT) images.

Nevertheless, a critical review of these studies reveals that they primarily rely on 2D image analyses. Therefore, a comprehensive 3D analysis that fully utilizes the volumetric information embedded within OCT scans to predict PDT response is still largely uncharted. Also, the application of DL to extrapolate treatment response from pre-treatment images, thereby providing an a priori estimate of treatment success, remains an area requiring further exploration. These gaps in current knowledge provide the motivation for the present study, urging us to propose a 3D DL approach for predicting PDT response in CSCR patients.

## Materials

### Datasets

In this study, we employed two custom datasets specifically designed for our research. The Ethics Committee of the Hospital Clí­nico San Carlos in Madrid (HCSC) approved the protocol of this study. The datasets for each task are detailed below.

#### 3D CSCR Fluid Segmentation Dataset

The dataset used for the segmentation task comprised 2769 OCT images where collected before PDT treatment from 124 persistent (SRF lasting more than 6 months) and complex (total area of RPE alteration greater than 2-disc area diameter) patients. Each of these images was paired with corresponding fluid labels, expertly annotated by an ophthalmologist with more than 10 years of experience. Data were organized into 124 3D volumes, each volume representing a distinct patient. The careful composition of this dataset ensured a diverse and representative assortment of OCT scans, comprising images from patients between 35 and 78 (mean age 55.2 Â± 8.2), both male (80.8%) and female (19.2%), and right and left eye images from a 54.8% and a 45.2% of the patients respectively, thereby enabling our model to effectively learn and generalize the task of fluid region segmentation.

#### Prediction of the Response to PDT Dataset

**Fig. 1:**
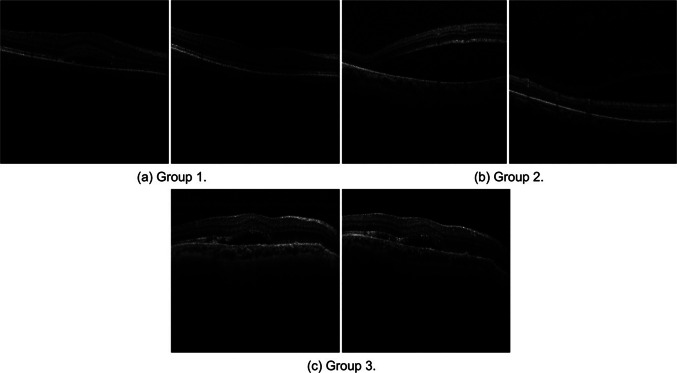
Illustrative example of pre- (1^st^ column) and post- (2^nd^ column) treatment images for a patient from each group. The presented images vividly portray the various degrees of SRF resolution following PDT treatment

The dataset used for the PDT response prediction task was composed of 216 volumes from 216 chronic CSCR patients treated with half-fluence PDT between January 2017 and December 2020 in the Hospital ClÃ­nico San Carlos, Madrid. Inclusion criteria were patients over 18 years old with diagnosed chronic CSCR that satisfied the major and minor criteria recently described for this disease [33], being all the cases persistent and complex, and eligible for half-fluence PDT, on the other hand patients with other retinal pathologies, suboptimal quality OCT images, severe retinal damage, or large photoreceptor atrophy were excluded. The patients were evaluated by experts based on the resolution of SRF 3 months after PDT treatment, this time point was set because it is when the response to PDT is assessed in clinical practice due to the progressive effect of this therapy that shows a higher rate of SRF resolution at 3 months than at 1 month. The main reason is that after PDT, it is frequent to observe an early worsening of the SRF related to the inflammation produced by the PDT [34]. The OCT software’s tools were used to measure and compare SRF before and 3 months after treatment, leading to the classification of patients into three groups:

- **Group 1**: 100 patients who exhibited complete resolution of SRF. Figure 1(a) illustrates the complete resorption of the SRF after treatment.
- **Group 2**: 66 patients who exhibited partial resolution of SRF, signified by at least a 15% decrease in baseline SRF height. As shown in Fig. 1(b), while the treatment led to a degree of SRF resorption, it was not fully resolved.
- **Group 3**: 50 patients who did not show any SRF resorption, where the decrease in baseline SRF height was less than 15%. Figure 1(c) demonstrates that despite the treatment, the SRF did not experience any significant reduction.

SD-OCT images were obtained using the Spectralis system from Heidelberg Engineering, Germany, both before and 3 months after PDT treatment. Macular cube images covering a 6 mm x 6 mm area were extracted from the OCT scans. The volume scan employed a “fast volume" preset, capturing 25 sections separated by 240 $\mu m$. Each section was averaged 9 times using automatic real-time (ART) technology, resulting in a lateral resolution of 512 pixels and an axial resolution of 496 pixels. Regarding patients information, ages ranged between 18 and 86 years, with a mean age of 55.6 Â± 10.8 years. As for the gender distribution, a 70.6% were males and a 29.4% females. Representative samples of pre- and post-treatment patient images from each group, exhibiting typical manifestations of CSCR, can be viewed in Fig. 1.

### Software and Hardware

In this research, Python (version 3.8.10) served as our primary programming language, due to its widespread use in scientific computing and the availability of numerous auxiliary libraries. For the segmentation task, we employed the nnU-Net library [35], which is well-regarded for its robust capabilities with 2D and 3D images. It is particularly suited to medical imaging tasks, given its ability to handle voxel spacings, anisotropies, and class imbalances.

To read and write 3D image data, we used the NiBabel library (version 4.0.2) [36], a Python package specifically designed for working with neuroimaging data. This tool was instrumental in efficiently handling our OCT scans.

The MONAI (Medical Open Network for AI) framework [37] was used for training and validation of the classification model in predicting PDT responses. This PyTorch-based platform is tailored for medical imaging applications, providing the necessary tools for developing our predictive model.

Performance evaluation of our classification model was facilitated by the Scikit-learn library [38], which offers robust methods for model evaluation, thereby helping us objectively assess our model’s performance.

Finally, all our models were trained, validated, and tested on a computer powered by an AMD Ryzen Threadripper 3960X 24-Core Processor and equipped with an NVIDIA® RTX A6000 GPU. This setup allowed us to efficiently conduct our computational tasks and run our experiments smoothly.

## Methodology

**Fig. 2:**
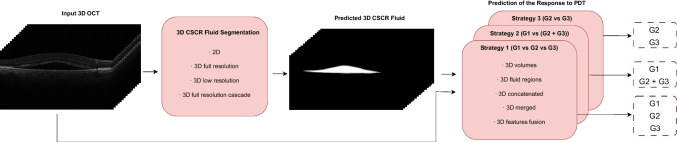
General scheme of the proposed methodology

In this section, we provide a detailed description of the methodology employed in this study. As shown in Fig. 2, the proposed system is designed to take a 3D image and perform the CSCR fluid segmentation, followed by the analisys of the PDT response through three different strategies. The following subsections provide more detailed information on the different components of the methodology, including the network architecture used for CSCR fluid segmentation, as well as the PDT response analysis approach.

### 3D CSCR Fluid Segmentation

**Fig. 3:**
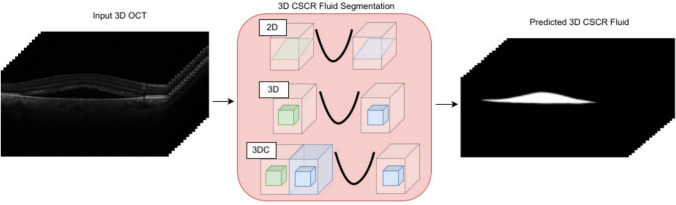
3D CSCR fluid segmentation pipeline

The accurate segmentation of the CSCR fluid is essential for the automated analysis of OCT images. To achieve this, we employed three 3D and a 2D configurations of the nnU-Net architecture to analyze their performance and determine which configuration produces better results. Figure 3 shows an overview of the 3D CSCR fluid segmentation pipeline, the input is a 3D OCT image and it outputs a 3D image with the 3D CSCR fluid region segmented. nnU-Net was selected as the reference architecture for semantic segmentation tasks in medical images due to its ability to accurately segment complex structures. These network architectures have been widely used in medical image segmentation tasks and have demonstrated the ability to accurately identify complex structures and objects within images [35]. By evaluating these architectures in our study, we aimed to determine the optimal approach for fluid segmentation in OCT images.

#### Network Architecture

In this work, we use nnU-Net that is an advanced DL architecture specifically designed for medical image segmentation tasks. It extends the original U-Net architecture on and follows an encoder-decoder structure and employs 3D U-Net modules, which serve as its core building blocks. These modules leverage 3D convolutions, max-pooling, and upsampling operations to extract high-level features from the input and generate accurate segmentation maps. The nnU-Net offers four different configurations:

- **2D:** processes 2D slices of medical images independently, making it computationally efficient but lacking the consideration of 3D spatial context between slices.
- **3D full resolution:** takes advantage of the entire 3D volume, preserving spatial relationships between slices for better accuracy, but it is computationally demanding and memory-intensive.
- **3D low resolution:** downsamples the 3D volume, striking a balance between efficiency and accuracy, while still retaining some spatial context.
- **3D full resolution cascade:** employs the segmentation results of the 3D low resolution configuration upsampled to the original voxel spacing and passed them as additional (one hot encoded) input channels to a 3D full resolution network, which is trained on patches at full resolution.

#### Training details

Regarding the training stage, we first randomly divided the used OCT image dataset into 2 smaller subsets, with 80% of cases for training and validation following a 5-fold cross-validation strategy and the remaining 20% for testing. It is also important to note that for each cross-validation fold a 80% and a 20% of cases were for training and validation respectively. In this way, we can obtain a more reliable and robust measure of the behaviour of the trained models. All images from one patient are exclusively assigned to one data set, avoiding any splits between the training, validation and testing data sets. This ensures the independence of the data sets and prevents any information leakage between them. The network parameters (weights and biases) are adjusted by applying the stochastic gradient descent (SGD) optimization method with a Nesterov momentum of 0.99 and an initial learning rate of 0.01. The learning rate is decaying according to the ’poly’ learning rate policy, $(1- \frac{epoch}{epoch_{max}})^{0.9}$. The training is conducted using a combination of Dice and Cross-Entropy as the loss function [39–41].

#### Data Augmentation

In recent years, DL has demonstrated very good performance on a variety of problems. However, to obtain robust and consistent results, it is necessary to train models with a considerable amount of data. In this way, an attempt is made to mitigate the well-known problem of over-fitting [42]. Therefore, in this work, we apply a data augmentation strategy to increase the size of the image dataset by applying different computer vision techniques. Specifically, we apply the data augmentation strategy, only on the training subset, using the following augmentation techniques on the fly during training: random rotations, random scaling, random elastic deformations, gamma correction augmentation and mirroring.

#### Evaluation

Precision, Recall, Accuracy, Jaccard and Dice coefficient are the most commonly used statistical metrics in the state of the art [22, 43] to quantitatively evaluate and validate the CSCR fluid segmentation developed by our method. These metrics are used to compare the predicted segmentation with the ground truth labels.

In addition to these metrics, we conducted repeated measures ANOVA analysis to further examine the performance of our segmentation method across different models. Furthermore, to provide deeper insights into the differences between segmentation results across different models, we performed a Tukey post hoc test. This test enabled us to identify specific pairwise differences in segmentation outcomes and determine statistically significant variations between groups. By employing repeated measures ANOVA analysis and Tukey post hoc test in conjunction with traditional evaluation metrics, we ensure a comprehensive assessment of the effectiveness and reliability of our method in the context of CSCR fluid segmentation.

### Prediction of the Response to PDT

Predicting a patient’s response to treatment before initiation is vital for effective management and recovery acceleration. For this study, we formulated three unique scenarios to extensively analyze the prediction of response to PDT using OCT images. Each scenario targets a different, clinically relevant condition. Detailed descriptions of these strategies are as follows:

- **Strategy 1: (Group 1) vs (Group 2) vs (Group 3)**. This first strategy examines the prediction of three distinct PDT responses in CSCR patients with persistent SRF: complete resorption, partial resorption, and the absence of any resorption. This comprehensive analysis evaluates the differentiability between all considered clinical scenarios.
- **Strategy 2: (Group 1) vs (Group 2 + Group 3)**. The second strategy adopts a predictive approach to assess the distinguishability between positive and negative PDT responses. To do this, we combine the responses of partial resorption and no resorption into a single class for analysis.
- **Strategy 3: (Group 2) vs (Group 3)**. The final strategy formulates a computational approach to determine the degree of separability between classes, focusing specifically on cases where the predicted response to PDT is negative.

These strategies aim to provide a comprehensive exploration and assessment of PDT response prediction in OCT images, considering various clinically significant scenarios.

#### 3D Computational Approaches for Prediction

**Fig. 4:**
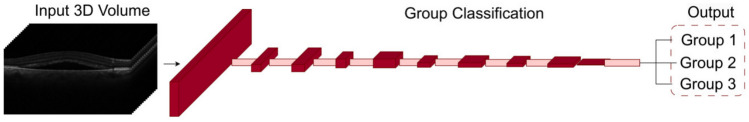
3D volumes method pipeline

**Fig. 5:**
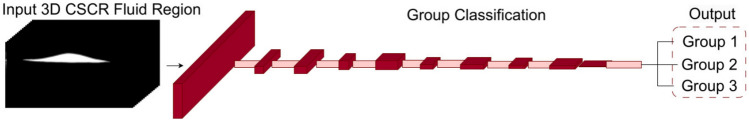
3D fluid regions method pipeline

**Fig. 6:**
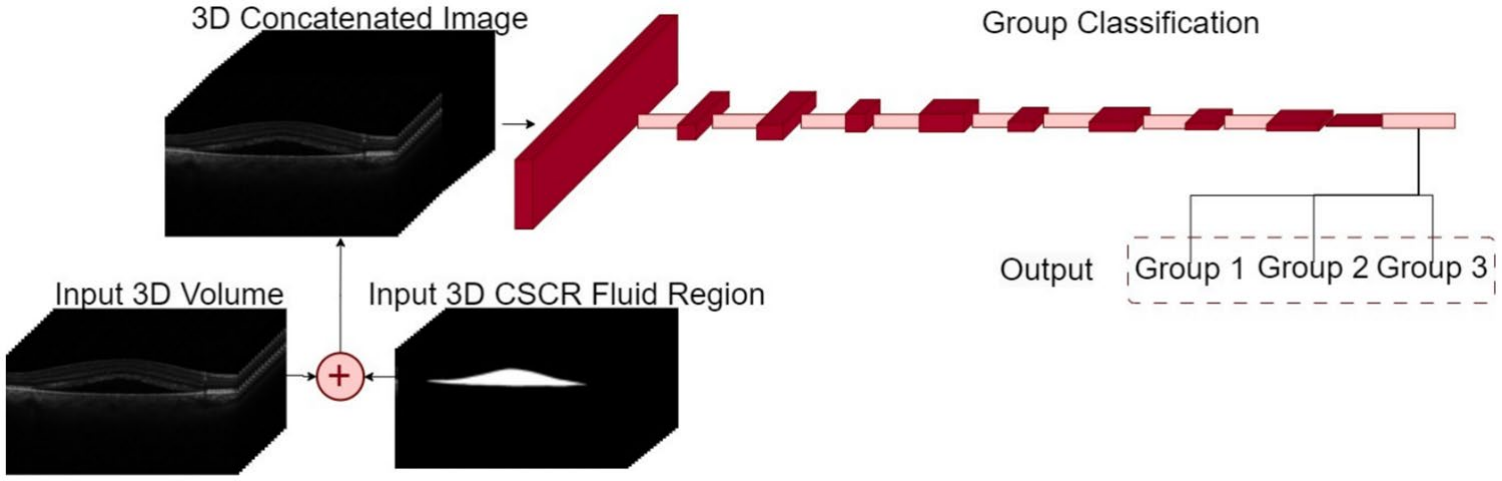
3D concatenated method pipeline

**Fig. 7:**
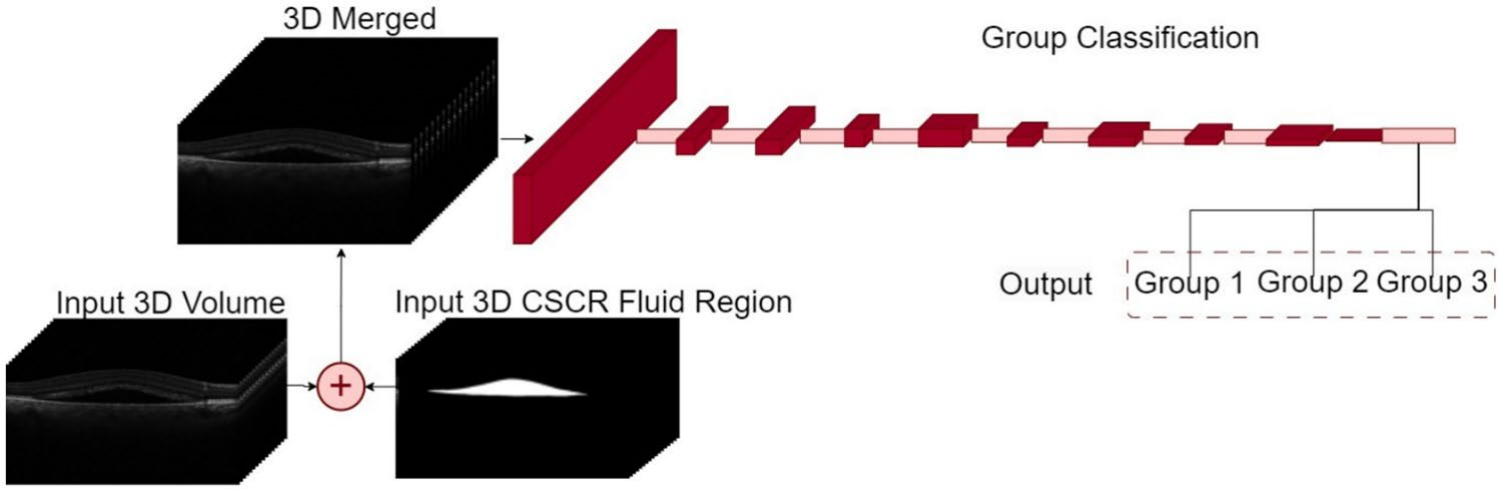
3D merged method pipeline

**Fig. 8:**
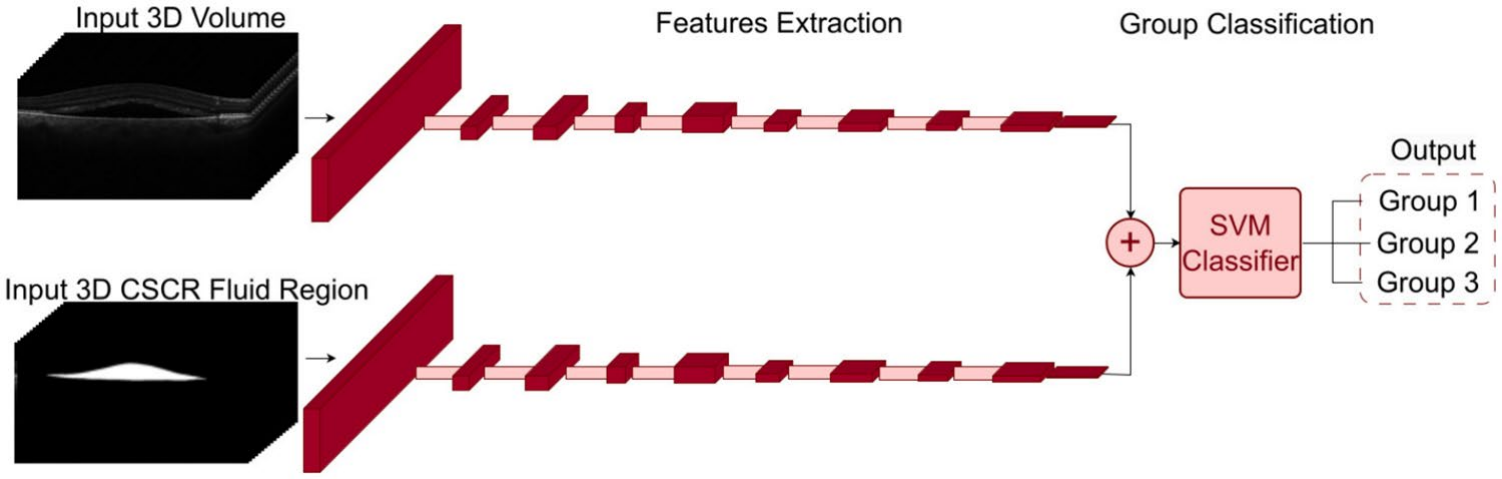
3D features fusion method pipeline

In our pursuit of achieving optimal classification performance, we have incorporated five distinct computational approaches in this study. The diversity in the use of input data by these approaches allows for a comprehensive exploration of their predictive capabilities with respect to our models. Each approach is outlined as follows:

- **3D volumes:** This approach employs a 3D DenseNet121 classifier, using 3D volumes as input to predict the treatment response group. See Fig. 4 for an overview of this method.
- **3D fluid regions:** This strategy, as shown in Fig. 5, utilizes the outputs of the 3D fluid segmentation method as inputs to a 3D DenseNet121 classifier.
- **3D concatenated:** This approach, visualized in Fig. 6, employs a 3D DenseNet121 classifier that uses concatenated 3D volumes and 3D fluid regions as input, predicting the corresponding group.
- **3D merged:** As depicted in Fig. 7, this method uses a 3D DenseNet121 classifier, taking an intercalated combination of 3D volumes and 3D fluid regions as input.
- **3D features fusion:** This strategy, outlined in Fig. 8, uses the previously trained classifiers to extract features from 3D volumes and 3D fluid regions. These features are then concatenated and a SVM classifier is used for final prediction.
- **3D features fusion + 2D Biomarkers:** For this approach, we extract the 2D Biomarkers and we concatenated it with the features obtained in the “3D features fusion” strategy. For the final prediction, we use the SVM classifier.
- **3D features fusion + 3D Biomarkers:** In this approach, we follow the same procedure as the previous one, but this time we replace the 2D biomarkers with their 3D counterparts in the concatenation process.
- **3D features fusion + 2D Biomarkers + 3D Biomarkers:** This strategy involves concatenating the features extracted from the "3D features fusion" approach with the 2D biomarkers calculated in "3D features fusion + 2D Biomarkers," and the 3D biomarkers obtained in "3D features fusion + 3D Biomarkers". The final prediction is made using a SVM classifier.

Each approach provides a unique perspective, enhancing our understanding of how varying forms of input data can impact the prediction of treatment response in OCT images.

#### Network architecture

DenseNet is a deep CNN architecture which has garnered popularity in the realm of medical image classification tasks, attributable to its unique design and exceptional performance. Introduced by Huang et al. in 2017 [44], it incorporates dense connections between layers, enabling each layer to receive the feature-maps of all preceding layers. This connectivity pattern promotes feature reuse, alleviates the vanishing gradient problem, and enhances the flow of information throughout the network. Specifically within the context of medical image classification, the dense connections of DenseNet facilitate efficient propagation of crucial features, empowering the network to discern intricate patterns and complex structures intrinsic to the images. Additionally, the compact and efficient design of DenseNet requires fewer parameters, making it an apt choice for medical image analysis tasks where data might be limited or imbalanced.

In this study, we have opted to utilize the 3D DenseNet-121 variant of DenseNet in four out of our five approaches (3D volumes, 3D fluid regions, 3D concatenated and 3D merged) for its proven efficiency and strong performance in handling 3D volumetric data, essential for the analysis and prediction tasks in our study.

**Fig. 9:**
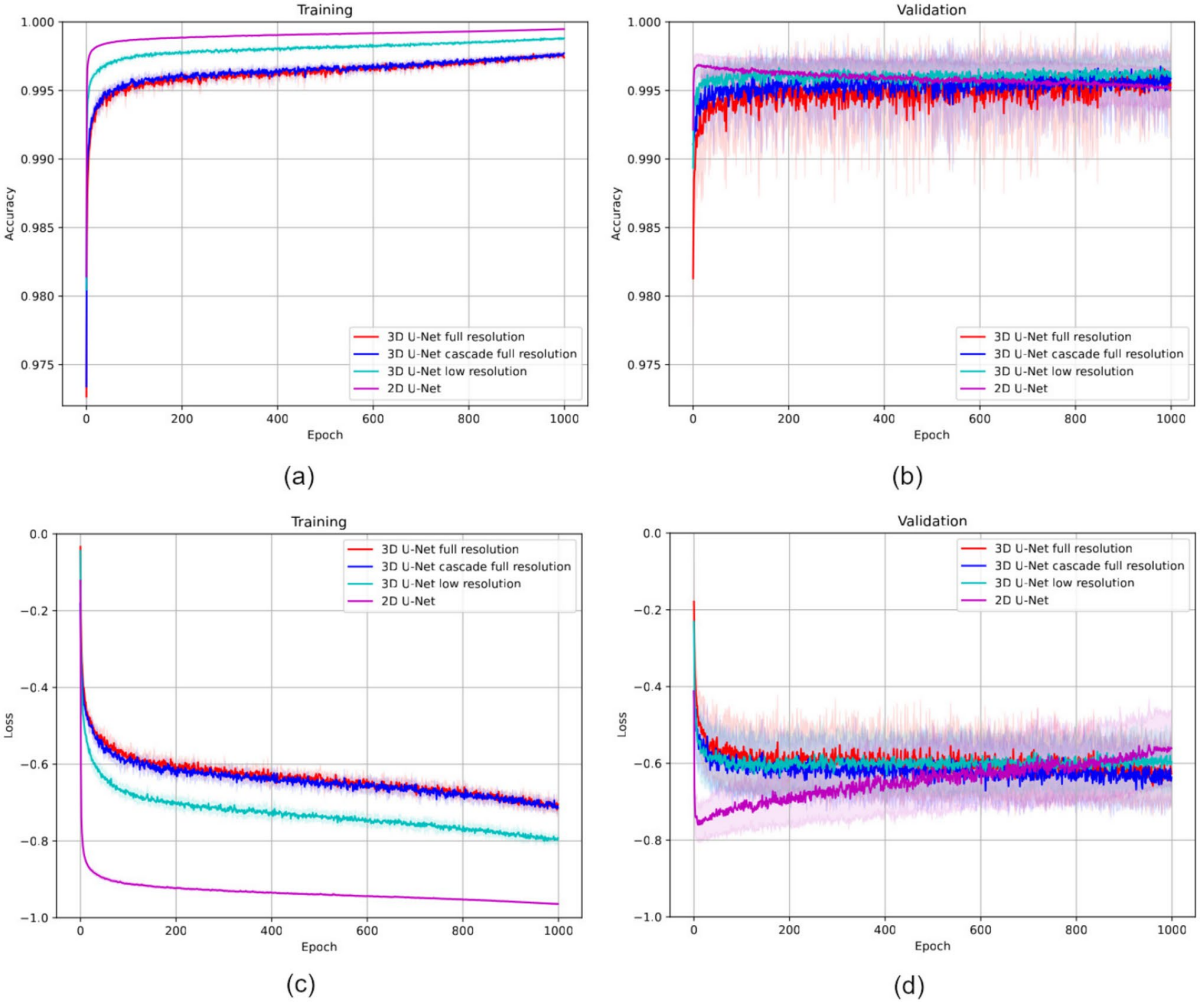
Evolution of the CSCR fluid segmentation trained models during the 5 folds in terms of mean ± standard deviation. **a** Train & **b** validation *Accuracy*, and **c** train & **d** validation *Loss*

#### 3D deep features extraction and classification

In the computational approach dubbed as “3D features fusion”, we employed 3D feature extraction and subsequent SVM classification [45], using our previously trained 3D DenseNet-121 models. These models facilitated extraction of deep features, which were then concatenated to fit and evaluate the SVM classifier.

The features were extracted from the output of the penultimate layer of the model, encapsulating the semantic information of the input images, hence providing more meaningful and compact representations compared to the raw pixel values. Two sets of 1,024 features were specifically obtained: one derived from the 3D volume and the other from the 3D CSCR fluid region. These two sets of features were concatenated post extraction, forming a comprehensive 2,048-feature vector.

Finally, this vector was fitted to a SVM classifier and its performance was evaluated. The choice of SVM classifier was motivated by its demonstrated effectiveness in medical imaging classification tasks [46–49].

#### Biomarkers Extraction

We obtained five different biomarkers from the 3D image, referred to as 3D Biomarkers. Additionally, we derived the same type of measurements from the 2D projection of the 3D image, dubbed as 2D Biomarkers. These five measurements were extracted for both 2D and 3D image modalities:

- **Area**: The area of the CSCR fluid region in pixels.
- **Area of the bounding box**: The area of the bounding box that encloses the CSCR fluid region in pixels.
- **Lenght of the major axis**: The length of the major axis of the ellipse that has the same normalized second central moments as the CSCR fluid region.
- **Lenght of the minor axis**: The minor axis length of the ellipse with the same the normalized second central moments as the CSCR fluid region.
- **Solidity**: The ratio of pixels in the CSCR fluid region to pixels of the smallest convex polygon that encloses the CSCR fluid region.

#### Training details

Our initial step involved randomly dividing the dataset in accordance with a 5-fold cross-validation scheme, wherein for each fold, 80% of the cases were used for training and the remaining 20% for validation. It is important to note that the same proportion of samples from each group as in the complete dataset was maintained. The adjustment of network parameters (weights and biases) was achieved through the Adam optimization method [50], with an initial learning rate of 0.001. The training was conducted utilizing Cross-Entropy as the loss function, with class weights adapted to address class imbalance. The advantage of using loss weights for class imbalance includes training balance by assigning higher weights to the minority class, leading to improved model performance and reduced biases. It stabilizes training, preserves information in severely imbalanced datasets, and allows control over the influence of each class on the overall loss function.

#### Evaluation

In assessing the effectiveness of our models in predicting PDT responses, we relied on established benchmarks in the realm of classification tasks: precision, recall, F1-score, and accuracy metrics. These metrics are ubiquitously employed by state-of-the-art models when tackling similar problems. We used repeated measures ANOVA analysis and Tukey post hoc test alongside traditional evaluation metrics to thoroughly assess the statistical differences between the test results obtained by the different classification approaches. These statistical methods helped identify significant differences in classification outcomes between approaches, ensuring a comprehensive evaluation of the effectiveness and reliability of our method in the prediction of the Response to PDT.

## Results and Discussion

This section covers our experimental setup and results designed to evaluate the proposed methodology. We have divided this section into three parts. First, we evaluate the 3D CSCR fluid segmentation process, and then we present the results of the PDT response analysis. Finally, we perform a comparison with existing literature.

### Evaluation of the 3D CSCR Fluid Segmentation

In our initial experiment, we focused on evaluating the effectiveness of our proposed method for CSCR fluid segmentation. Additionally, we compared the performance of the nnU-Net architecture using four different configurations for both 2D and 3D segmentation. To conduct a robust assessment, we employed 5-fold cross-validation, ensuring reliable results. The progression during training and validation stages is depicted in Fig. 9. The outcomes reveal that, on the whole, all 3D configurations exhibit satisfactory CSCR fluid segmentation in OCT images. Notably, these configurations achieve stability from epoch 950, as evidenced by consistent mean and standard deviation values. However, the 2D nnU-Net approach demonstrated an interesting pattern. While it initially attains high training accuracy in the early epochs, it subsequently falls victim to overfitting. As the model grows increasingly complex, it starts to memorize noise and concrete features present in the training data, leading to a loss of its generalization capabilities.

**Fig. 10:**
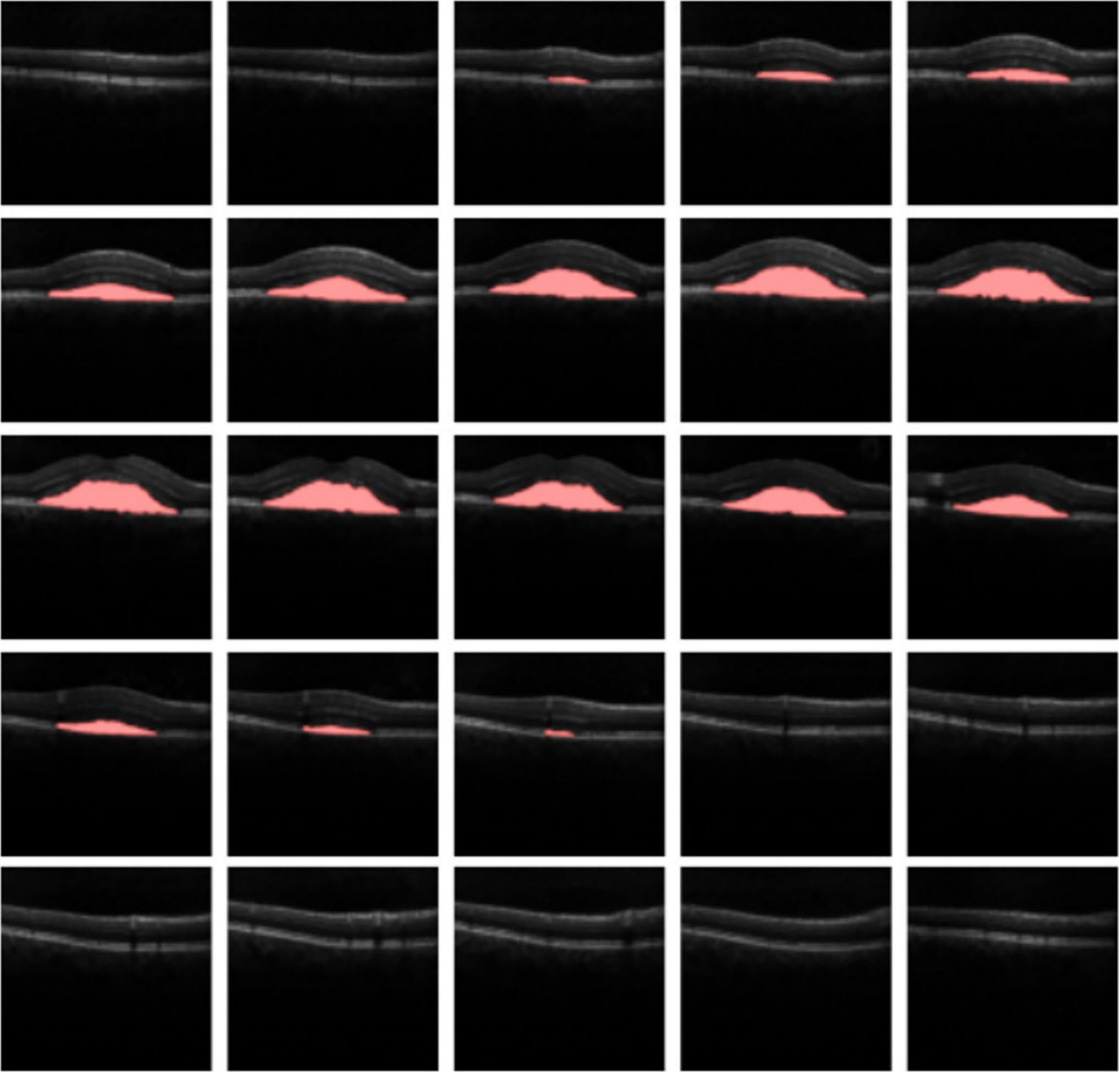
Representation of the segmented CSCR fluid region over the correspondent input image

Table 1 presents the test results obtained from 5-fold cross-validation, represented by mean values and their corresponding standard deviations. The evaluation aims to assess the performance of our proposed CSCR fluid segmentation system using different configurations.The results indicate that our proposed system achieves satisfactory performance for the full resolution configurations on the test subsets. Specifically, the 3D nnU-Net with full resolution achieves the best results, demonstrating robustness in fluid segmentation. The average Precision is $0.8267\pm 0.2564$, the average Recall is $0.7335\pm 0.2283$, the average Accuracy is $0.9975\pm 0.0027$, the average Jaccard Index is $0.6435\pm 0.2534$, and the average Dice Coefficient is $0.7448\pm 0.2494$. In contrast, the 2D nnU-Net and 3D low-resolution configurations show lower performance metrics, likely due to the loss of 3D information and the loss of information during resolution reduction, respectively. These observations underscore the importance of leveraging the full resolution and 3D information for improved segmentation results in OCT images. We perform a repeated measures ANOVA analysis in order to analyse statistically for significant differences comparing the 4 architectures. We found significant differences at $\alpha =0.05$ for all the metrics presenting all of them a $p-value < 0.0001$, except Precision with a $p-value=0.0311$. We also perform Tukey post hoc test, whose results can be found in Table 2. Figure 10 is an illustrative example of the resulting segmented CSCR region over the input image.

**Table 1:** Mean ± standard deviation of CSCR fluid segmentation test results for the 5 folds

***Architecture***	***Accuracy***	***Jaccard***	***Dice***	***Precision***	***Recall***
**2d-overall**	$0.9959\pm 0.0038$	$0.4304\pm 0.3013$	$0.5367\pm 0.3107$	$0.8098\pm 0.2636$	$0.4971\pm 0.3194$
**3d-fullres-overall**	$0.9975\pm 0.0027$	$0.6435\pm 0.2534$	$0.7448\pm 0.2494$	$0.8267\pm 0.2564$	$0.7335\pm 0.2283$
**3d-lowres-overall**	$0.9957\pm 0.0035$	$0.4446\pm 0.2568$	$0.5698\pm 0.2610$	$0.7364\pm 0.2518$	$0.5470\pm 0.2703$
**3d-cascade-fullres-overall**	$0.9976\pm 0.0026$	$0.6270\pm 0.2643$	$0.7266\pm 0.2739$	$0.7800\pm 0.2920$	$0.7643\pm 0.2158$

**Table 2:** Tukey post hoc test results for pairwise comparison between the test results of the segmentation models. Comparisons with no statistically significant difference ($p-value>=0.05$) are represented by $\approx$ and $\Uparrow$ represent the situations where there is a statistically significant difference ($p-value<0.05$)

***Architecture 1***	**Architecture 2**	**Accuracy**	**Jaccard**	**Dice**	**Precision**	**Recall**
**2d-overall**	**3d-fullres-overall**	$\Uparrow$	$\Uparrow$	$\Uparrow$	$\approx$	$\Uparrow$
**2d-overall**	**3d-lowres-overall**	$\approx$	$\approx$	$\approx$	$\approx$	$\Uparrow$
**2d-overall**	**3d-cascade-fullres-overall**	$\Uparrow$	$\Uparrow$	$\Uparrow$	$\approx$	$\Uparrow$
**3d-fullres-overall**	**3d-lowres-overall**	$\Uparrow$	$\Uparrow$	$\Uparrow$	$\Uparrow$	$\Uparrow$
**3d-fullres-overall**	**3d-cascade-fullres-overall**	$\approx$	$\approx$	$\approx$	$\approx$	$\approx$
**3d-lowres-overall**	**3d-cascade-fullres-overall**	$\Uparrow$	$\Uparrow$	$\Uparrow$	$\approx$	$\Uparrow$

### Evaluation of the Prediction of the Response to PDT

This subsection focuses on evaluate the predictive performance of different approaches in determining the response to PDT in OCT images.

#### Predictive analysis of Group 1 vs. Group 2 vs. Group 3

In this first strategy we have analyzed 216 cases, 100 belong to group 1, 66 to group 2 and 50 to group 3. Table 3 show the results of the different experiments developed. The best precision was found using 3D features fusion as input, achieving a mean accuracy value of $0.6438\pm 0.0617$, as well as a $0.7507\pm 0.0442$ group 1 precision value. Regarding the other methods, we observe that 3D merged performs worse than 3D volumes, so that this fusion of data is not appropriated for this task. We have performed a repeated measures ANOVA analysis with $\alpha =0.05$. In this analysis we found significant differences between the results obtained by the different approaches with $p-value 0.0078$ for all metrics, except for group 1 recall ($p-value=0.5706$), that no significant differences were found. These findings suggest notable variations in outcomes across different classification approaches. Moreover, Table 4 outlines the results of the Tukey post hoc test, illustrating significant differences between approaches incorporating 3D feature fusion and those that do not.

**Table 3:** PDT response prediction classification results for strategy 1 (G1 vs G2 vs G3)

**Input**	**Group**	**Precision**	**Recall**	**F1-score**	**Accuracy**
3D volumes	1	$0.6505\pm 0.1000$	$0.7200\pm 0.1860$	$0.6593\pm 0.0682$	$0.5832\pm 0.0596$
	2	$0.7000\pm 0.1753$	$0.5879\pm 0.2340$	$0.5698\pm 0.1565$	
	3	$0.3509\pm 0.1902$	$0.3000\pm 0.1673$	$0.3191\pm 0.1707$	
3D fluid regions	1	$0.5847\pm 0.0382$	$0.8300\pm 0.1122$	$0.6812\pm 0.0407$	$0.5690\pm 0.0507$
	2	$0.6512\pm 0.1414$	$0.4231\pm 0.1614$	$0.4908\pm 0.1139$	
	3	$0.4245\pm 0.2629$	$0.2400\pm 0.1200$	$0.2979\pm 0.1564$	
3D concatenated	1	$0.6095\pm 0.1037$	$0.9100\pm 0.0490$	$0.7217\pm 0.0605$	$0.5927\pm 0.0846$
	2	$0.5351\pm 0.2762$	$0.3791\pm 0.2530$	$0.4242\pm 0.2461$	
	3	$0.5083\pm 0.3296$	$0.2400\pm 0.1625$	$0.2949\pm 0.1626$	
3D merged	1	$0.6805\pm 0.1133$	$0.7000\pm 0.2387$	$0.6479\pm 0.0926$	$0.5695\pm 0.0743$
	2	$0.4403\pm 0.2309$	$0.5407\pm 0.2902$	$0.4752\pm 0.2394$	
	3	$0.5495\pm 0.2510$	$0.3400\pm 0.2245$	$0.3574\pm 0.1855$	
3D features fusion	1	$0.7507\pm 0.0442$	$0.6600\pm 0.1655$	$0.6952\pm 0.1137$	$0.6438\pm 0.0617$
	2	$0.6254\pm 0.0417$	$0.6220\pm 0.0791$	$0.6201\pm 0.0384$	
	3	$0.5189\pm 0.1153$	$0.6400\pm 0.1020$	$0.5701\pm 0.1060$	
3D features fusion + 2D biomarkers	1	$0.7507\pm 0.0442$	$0.6600\pm 0.1655$	$0.6952\pm 0.1137$	$0.6344\pm 0.0624$
	2	$0.6056\pm 0.0352$	$0.5901\pm 0.0813$	$0.5927\pm 0.0357$	
	3	$0.5114\pm 0.1172$	$0.6400\pm 0.1020$	$0.5657\pm 0.1078$	
3D features fusion + 3D biomarkers	1	$0.7595\pm 0.0543$	$0.6600\pm 0.1655$	$0.6992\pm 0.1166$	$0.6437\pm 0.0691$
	2	$0.6250\pm 0.0261$	$0.6209\pm 0.0850$	$0.6182\pm 0.0407$	
	3	$0.5114\pm 0.1197$	$0.6400\pm 0.1020$	$0.5660\pm 0.1106$	
3D features fusion + 2D biomarkers + 3D biomarkers	1	$0.7595\pm 0.0543$	$0.6600\pm 0.1655$	$0.6992\pm 0.1166$	$0.6390\pm 0.0692$
	2	$0.6186\pm 0.0311$	$0.6055\pm 0.0913$	$0.6071\pm 0.0470$	
	3	$0.5037\pm 0.1190$	$0.6400\pm 0.1020$	$0.5609\pm 0.1091$

**Table 4:** Tukey post hoc test results for pairwise comparison between the test results of the classification approaches for strategy 1 (G1 vs G2 vs G3). Comparisons with no statistically significant difference ($p-value>=0.05$) are represented by $\approx$ and $\Uparrow$ represent the situations where there is a statistically significant difference ($p-value<0.05$)

***Input 1***	***Input 2***	***Acc.***	***Prec. G1***	***Prec. G2***	***Prec. G3***	***Rec. G1***	***Rec. G2***	***Rec. G3***	***F1 G1***	***F1 G2***	***F1 G3***
**3D volumes**	**3D fluid regions**	$\approx$	$\approx$	$\approx$	$\approx$	$\approx$	$\approx$	$\approx$	$\approx$	$\approx$	$\approx$
**3D volumes**	**3D concatenated**	$\approx$	$\approx$	$\approx$	$\approx$	$\approx$	$\approx$	$\approx$	$\approx$	$\approx$	$\approx$
**3D volumes**	**3D merged**	$\approx$	$\approx$	$\approx$	$\approx$	$\approx$	$\approx$	$\approx$	$\approx$	$\approx$	$\approx$
**3D volumes**	**3D features fusion**	$\Uparrow$	$\approx$	$\approx$	$\approx$	$\approx$	$\approx$	$\Uparrow$	$\approx$	$\approx$	$\Uparrow$
**3D volumes**	**3D features fusion+2D biomarkers**	$\Uparrow$	$\approx$	$\approx$	$\approx$	$\approx$	$\approx$	$\Uparrow$	$\approx$	$\approx$	$\approx$
**3D volumes**	**3D features fusion+3D biomarkers**	$\Uparrow$	$\approx$	$\approx$	$\approx$	$\approx$	$\approx$	$\Uparrow$	$\approx$	$\approx$	$\Uparrow$
**3D volumes**	**3D features fusion+2D biomarkers+3D biomarkers**	$\Uparrow$	$\approx$	$\approx$	$\approx$	$\approx$	$\approx$	$\Uparrow$	$\approx$	$\approx$	$\approx$
**3D fluid regions**	**3D concatenated**	$\approx$	$\approx$	$\approx$	$\approx$	$\approx$	$\approx$	$\approx$	$\approx$	$\approx$	$\approx$
**3D fluid regions**	**3D merged**	$\approx$	$\approx$	$\approx$	$\approx$	$\approx$	$\approx$	$\approx$	$\approx$	$\approx$	$\approx$
**3D fluid regions**	**3D features fusion**	$\Uparrow$	$\approx$	$\approx$	$\Uparrow$	$\approx$	$\approx$	$\Uparrow$	$\Uparrow$	$\Uparrow$	$\Uparrow$
**3D fluid regions**	**3D features fusion+2D biomarkers**	$\Uparrow$	$\approx$	$\approx$	$\Uparrow$	$\approx$	$\approx$	$\Uparrow$	$\Uparrow$	$\Uparrow$	$\Uparrow$
**3D fluid regions**	**3D features fusion+3D biomarkers**	$\Uparrow$	$\approx$	$\approx$	$\Uparrow$	$\approx$	$\approx$	$\Uparrow$	$\Uparrow$	$\Uparrow$	$\Uparrow$
**3D fluid regions**	**3D features fusion+2D biomarkers+3D biomarkers**	$\Uparrow$	$\approx$	$\approx$	$\approx$	$\approx$	$\approx$	$\Uparrow$	$\Uparrow$	$\Uparrow$	$\Uparrow$
**3D concatenated**	**3D merged**	$\approx$	$\approx$	$\approx$	$\approx$	$\approx$	$\approx$	$\approx$	$\approx$	$\approx$	$\approx$
**3D concatenated**	**3D features fusion**	$\Uparrow$	$\Uparrow$	$\Uparrow$	$\approx$	$\approx$	$\Uparrow$	$\Uparrow$	$\Uparrow$	$\Uparrow$	$\Uparrow$
**3D concatenated**	**3D features fusion+2D biomarkers**	$\Uparrow$	$\Uparrow$	$\Uparrow$	$\approx$	$\approx$	$\Uparrow$	$\Uparrow$	$\approx$	$\Uparrow$	$\Uparrow$
**3D concatenated**	**3D features fusion+3D biomarkers**	$\Uparrow$	$\Uparrow$	$\Uparrow$	$\approx$	$\approx$	$\Uparrow$	$\Uparrow$	$\approx$	$\Uparrow$	$\Uparrow$
**3D concatenated**	**3D features fusion+2D biomarkers+3D biomarkers**	$\Uparrow$	$\Uparrow$	$\Uparrow$	$\approx$	$\approx$	$\Uparrow$	$\Uparrow$	$\approx$	$\Uparrow$	$\Uparrow$
**3D merged**	**3D features fusion**	$\Uparrow$	$\approx$	$\Uparrow$	$\approx$	$\approx$	$\approx$	$\approx$	$\Uparrow$	$\Uparrow$	$\approx$
**3D merged**	**3D features fusion+2D biomarkers**	$\Uparrow$	$\approx$	$\approx$	$\approx$	$\approx$	$\approx$	$\approx$	$\Uparrow$	$\Uparrow$	$\approx$
**3D merged**	**3D features fusion+3D biomarkers**	$\Uparrow$	$\approx$	$\Uparrow$	$\approx$	$\approx$	$\approx$	$\approx$	$\Uparrow$	$\Uparrow$	$\approx$
**3D merged**	**3D features fusion+2D biomarkers+3D biomarkers**	$\Uparrow$	$\approx$	$\Uparrow$	$\approx$	$\approx$	$\approx$	$\approx$	$\Uparrow$	$\Uparrow$	$\approx$
**3D features fusion**	**3D features fusion+2D biomarkers**	$\approx$	$\approx$	$\approx$	$\approx$	$\approx$	$\approx$	$\approx$	$\approx$	$\approx$	$\approx$
**3D features fusion**	**3D features fusion+3D biomarkers**	$\approx$	$\approx$	$\approx$	$\approx$	$\approx$	$\approx$	$\approx$	$\approx$	$\approx$	$\approx$
**3D features fusion**	**3D features fusion+2D biomarkers+3D biomarkers**	$\approx$	$\approx$	$\approx$	$\approx$	$\approx$	$\approx$	$\approx$	$\approx$	$\approx$	$\approx$
**3D features fusion+2D biomarkers**	**3D features fusion+3D biomarkers**	$\approx$	$\approx$	$\approx$	$\approx$	$\approx$	$\approx$	$\approx$	$\approx$	$\approx$	$\approx$
**3D features fusion+2D biomarkers**	**3D features fusion+2D biomarkers+3D biomarkers**	$\approx$	$\approx$	$\approx$	$\approx$	$\approx$	$\approx$	$\approx$	$\approx$	$\approx$	$\approx$
**3D features fusion+3D biomarkers**	**3D features fusion+2D biomarkers+3D biomarkers**	$\approx$	$\approx$	$\approx$	$\approx$	$\approx$	$\approx$	$\approx$	$\approx$	$\approx$	$\approx$

#### Predictive analysis of Group 1 vs. Group 2 & Group 3

In this second strategy, we analyze 100 eyes of group 1 and 116 included in groups 2 and 3. The outcomes of this experiment are presented in Table 5. The best metric values were obtained with the 3D features fusion, reaching a mean accuracy value of $0.7923\pm 0.0942$ and a F1-score for the groups 2 + 3 of $0.8034\pm 0.0839$. For these strategy, performing the 3D merged fusion information improves slightly the results of the one input methods. We conducted a repeated measures ANOVA analysis ($\alpha =0.05$), revealing a $p-value < 0.0001$ across all classification metrics except for precision for the group 1 ($p-value = 0.0104$) and precision for the groups 2 + 3, that is the only metric that do not showed significant differences with a $p-value = 0.2370$. These findings underscore substantial disparities among the outcomes generated by the different classification methods. Furthermore, Table 6 presents the results of the Tukey post hoc test, emphasizing notable distinctions between approaches incorporating 3D feature fusion and those that do not, except for 3D-volumes.

#### Predictive analysis of Group 2 vs. Group 3

In this third strategy, we examined 66 volumes belonging to group 2 and 50 volumes included in group 3. The results obtained for this strategy are presented in Table 7. The mean accuracy value reached $0.9141\pm 0.0608$, and the recall value for group 2 was $0.9407\pm 0.0728$. This method outperforms the other ones by approximately a 20%. In this particular case, the fusion of the 3D volumes data results in a worse classification.We have performed a repeated measures ANOVA analysis ($\alpha =0.05$), in this analysis we obtain a $p-value < 0.0001$ for all the classification metrics, except a $p-value = 0.0044$ for group 2 recall. These results showed that there are significant differences between the results obtained by the different classification approaches. In adition, in Table 8 we show the results of the Tukey post hoc test, that highlights that significant differences are found between the approaches that involve 3D features fusion and the ones that do not involve them.

**Table 5:** PDT response prediction classification results for strategy 2 (G1 vs (G2 + G3))

**Input**	**Group**	**Precision**	**Recall**	**F1-score**	**Accuracy**
3D volumes	1	$0.7540\pm 0.0563$	$0.5300\pm 0.2040$	$0.5968\pm 0.1459$	$0.6988\pm 0.0616$
	2 + 3	$0.6910\pm 0.0784$	$0.8442\pm 0.0901$	$0.7517\pm 0.0337$	
3D fluid regions	1	$0.6379\pm 0.0190$	$0.6800\pm 0.1288$	$0.6508\pm 0.0626$	$0.6712\pm 0.0233$
	2 + 3	$0.7178\pm 0.0608$	$0.6649\pm 0.0779$	$0.6834\pm 0.0198$	
3D concatenated	1	$0.7815\pm 0.0796$	$0.5900\pm 0.1715$	$0.6505\pm 0.1390$	$0.7266\pm 0.0671$
	2 + 3	$0.7136\pm 0.0652$	$0.8446\pm 0.0940$	$0.7685\pm 0.0514$	
3D merged	1	$0.6087\pm 0.0331$	$0.7900\pm 0.0663$	$0.6864\pm 0.0385$	$0.6667\pm 0.0401$
	2 + 3	$0.7591\pm 0.0599$	$0.5601\pm 0.0648$	$0.6420\pm 0.0528$	
3D features fusion	1	$0.7646\pm 0.1095$	$0.8000\pm 0.1378$	$0.7781\pm 0.1074$	$0.7923\pm 0.0942$
	2 + 3	$0.8296\pm 0.1103$	$0.7851\pm 0.0931$	$0.8034\pm 0.0839$	
3D features fusion + 2D biomarkers	1	$0.7582\pm 0.1176$	$0.8000\pm 0.1378$	$0.7750\pm 0.1122$	$0.7877\pm 0.1008$
	2 + 3	$0.8270\pm 0.1140$	$0.7768\pm 0.1011$	$0.7980\pm 0.0914$	
3D features fusion + 3D biomarkers	1	$0.7541\pm 0.0934$	$0.8000\pm 0.1378$	$0.7735\pm 0.1041$	$0.7876\pm 0.0900$
	2 + 3	$0.8284\pm 0.1102$	$0.7764\pm 0.0773$	$0.7989\pm 0.0791$	
3D features fusion + 2D biomarkers + 3D biomarkers	1	$0.7399\pm 0.1068$	$0.7900\pm 0.1281$	$0.7619\pm 0.1075$	$0.7738\pm 0.0978$
	2 + 3	$0.8137\pm 0.1072$	$0.7594\pm 0.0946$	$0.7836\pm 0.0902$

**Table 6:** Tukey post hoc test results for pairwise comparison between the test results of the classification approaches for strategy 2 (G1 vs (G2 + G3)). Comparisons with no statistically significant difference ($p-value>=0.05$) are represented by $\approx$ and $\Uparrow$ represent the situations where there is a statistically significant difference ($p-value<0.05$)

***Input 1***	***Input 2***	***Acc.***	***Prec. G1***	***Prec. (G2 + G3)***	***Rec. G1***	***Rec. (G2 + G3)***	***F1 G1***	***F1 (G2 + G3)***
**3D volumes**	**3D fluid regions**	$\Uparrow$	$\approx$	$\approx$	$\Uparrow$	$\Uparrow$	$\approx$	$\Uparrow$
**3D volumes**	**3D concatenated**	$\approx$	$\approx$	$\approx$	$\approx$	$\approx$	$\approx$	$\approx$
**3D volumes**	**3D merged**	$\approx$	$\approx$	$\approx$	$\approx$	$\approx$	$\approx$	$\approx$
**3D volumes**	**3D features fusion**	$\Uparrow$	$\approx$	$\approx$	$\approx$	$\approx$	$\Uparrow$	$\approx$
**3D volumes**	**3D features fusion+2D biomarkers**	$\approx$	$\approx$	$\approx$	$\approx$	$\approx$	$\Uparrow$	$\approx$
**3D volumes**	**3D features fusion+3D biomarkers**	$\approx$	$\approx$	$\approx$	$\approx$	$\approx$	$\approx$	$\approx$
**3D volumes**	**3D features fusion+2D biomarkers+3D biomarkers**	$\approx$	$\approx$	$\approx$	$\approx$	$\approx$	$\approx$	$\approx$
**3D fluid regions**	**3D concatenated**	$\approx$	$\approx$	$\approx$	$\Uparrow$	$\Uparrow$	$\approx$	$\Uparrow$
**3D fluid regions**	**3D merged**	$\approx$	$\approx$	$\approx$	$\Uparrow$	$\Uparrow$	$\approx$	$\Uparrow$
**3D fluid regions**	**3D features fusion**	$\Uparrow$	$\approx$	$\approx$	$\approx$	$\Uparrow$	$\approx$	$\Uparrow$
**3D fluid regions**	**3D features fusion+2D biomarkers**	$\Uparrow$	$\approx$	$\approx$	$\approx$	$\Uparrow$	$\approx$	$\Uparrow$
**3D fluid regions**	**3D features fusion+3D biomarkers**	$\Uparrow$	$\approx$	$\approx$	$\approx$	$\Uparrow$	$\approx$	$\Uparrow$
**3D fluid regions**	**3D features fusion+2D biomarkers+3D biomarkers**	$\Uparrow$	$\approx$	$\approx$	$\approx$	$\Uparrow$	$\approx$	$\Uparrow$
**3D concatenated**	**3D merged**	$\approx$	$\approx$	$\approx$	$\approx$	$\approx$	$\approx$	$\approx$
**3D concatenated**	**3D features fusion**	$\Uparrow$	$\approx$	$\approx$	$\Uparrow$	$\approx$	$\Uparrow$	$\approx$
**3D concatenated**	**3D features fusion+2D biomarkers**	$\Uparrow$	$\approx$	$\approx$	$\Uparrow$	$\approx$	$\Uparrow$	$\approx$
**3D concatenated**	**3D features fusion+3D biomarkers**	$\Uparrow$	$\approx$	$\approx$	$\Uparrow$	$\approx$	$\Uparrow$	$\approx$
**3D concatenated**	**3D features fusion+2D biomarkers+3D biomarkers**	$\Uparrow$	$\approx$	$\approx$	$\Uparrow$	$\approx$	$\Uparrow$	$\approx$
**3D merged**	**3D features fusion**	$\Uparrow$	$\approx$	$\approx$	$\approx$	$\approx$	$\Uparrow$	$\Uparrow$
**3D merged**	**3D features fusion+2D biomarkers**	$\Uparrow$	$\approx$	$\approx$	$\approx$	$\approx$	$\Uparrow$	$\approx$
**3D merged**	**3D features fusion+3D biomarkers**	$\Uparrow$	$\approx$	$\approx$	$\approx$	$\approx$	$\Uparrow$	$\approx$
**3D merged**	**3D features fusion+2D biomarkers+3D biomarkers**	$\Uparrow$	$\approx$	$\approx$	$\approx$	$\approx$	$\Uparrow$	$\approx$
**3D features fusion**	**3D features fusion+2D biomarkers**	$\approx$	$\approx$	$\approx$	$\approx$	$\approx$	$\approx$	$\approx$
**3D features fusion**	**3D features fusion+3D biomarkers**	$\approx$	$\approx$	$\approx$	$\approx$	$\approx$	$\approx$	$\approx$
**3D features fusion**	**3D features fusion+2D biomarkers+3D biomarkers**	$\approx$	$\approx$	$\approx$	$\approx$	$\approx$	$\approx$	$\approx$
**3D features fusion+2D biomarkers**	**3D features fusion+3D biomarkers**	$\approx$	$\approx$	$\approx$	$\approx$	$\approx$	$\approx$	$\approx$
**3D features fusion+2D biomarkers**	**3D features fusion+2D biomarkers+3D biomarkers**	$\approx$	$\approx$	$\approx$	$\approx$	$\approx$	$\approx$	$\approx$
**3D features fusion+3D biomarkers**	**3D features fusion+2D biomarkers+3D biomarkers**	$\approx$	$\approx$	$\approx$	$\approx$	$\approx$	$\approx$	$\approx$

**Table 7:** PDT response prediction classification results for strategy 3 (G2 vs G3)

**Input**	**Group**	**Precision**	**Recall**	**F1-score**	**Accuracy**
3D volumes	2	$0.7540\pm 0.0971$	$0.8308\pm 0.1231$	$0.7795\pm 0.0651$	$0.7330\pm 0.0834$
	3	$0.7631\pm 0.1465$	$0.6000\pm 0.2449$	$0.6314\pm 0.1693$	
3D fluid regions	2	$0.8550\pm 0.1249$	$0.6978\pm 0.1591$	$0.7482\pm 0.0640$	$0.7417\pm 0.0599$
	3	$0.7014\pm 0.1509$	$0.8000\pm 0.1789$	$0.7230\pm 0.0702$	
3D concatenated	2	$0.7200\pm 0.0313$	$0.8308\pm 0.1492$	$0.7629\pm 0.0601$	$0.7156\pm 0.0436$
	3	$0.7820\pm 0.1526$	$0.5600\pm 0.1497$	$0.6191\pm 0.0836$	
3D merged	2	$0.7440\pm 0.0389$	$0.8011\pm 0.1057$	$0.7648\pm 0.0352$	$0.7243\pm 0.0186$
	3	$0.7231\pm 0.0624$	$0.6200\pm 0.1327$	$0.6525\pm 0.0612$	
3D features fusion	2	$0.9129\pm 0.0539$	$0.9407\pm 0.0728$	$0.9253\pm 0.0540$	$0.9141\pm 0.0608$
	3	$0.9236\pm 0.0937$	$0.8800\pm 0.0748$	$0.8987\pm 0.0696$	
3D features fusion + 2D biomarkers	2	$0.9272\pm 0.0646$	$0.9407\pm 0.0728$	$0.9327\pm 0.0608$	$0.9228\pm 0.0688$
	3	$0.9236\pm 0.0937$	$0.9000\pm 0.0894$	$0.9092\pm 0.0794$	
3D features fusion + 3D biomarkers	2	$0.9283\pm 0.0645$	$0.9549\pm 0.0610$	$0.9407\pm 0.0570$	$0.9312\pm 0.0649$
	3	$0.9400\pm 0.0800$	$0.9000\pm 0.0894$	$0.9178\pm 0.0756$	
3D features fusion + 2D biomarkers + 3D biomarkers	2	$0.9272\pm 0.0646$	$0.9407\pm 0.0728$	$0.9327\pm 0.0608$	$0.9228\pm 0.0688$
	3	$0.9236\pm 0.0937$	$0.9000\pm 0.0894$	$0.9092\pm 0.0794$

**Table 8:** Tukey post hoc test results for pairwise comparison between the test results of the classification approaches for strategy 3 (G2 vs G3). Comparisons with no statistically significant difference ($p-value>=0.05$) are represented by $\approx$ and $\Uparrow$ represent the situations where there is a statistically significant difference ($p-value<0.05$)

***Input 1***	***Input 2***	***Acc.***	***Prec. G2***	***Prec. G3***	***Rec. G2***	***Rec. G3***	***F1 G2***	***F1 G3***
**3D volumes**	**3D fluid regions**	$\approx$	$\approx$	$\Uparrow$	$\approx$	$\approx$	$\approx$	$\Uparrow$
**3D volumes**	**3D concatenated**	$\approx$	$\approx$	$\approx$	$\approx$	$\approx$	$\approx$	$\approx$
**3D volumes**	**3D merged**	$\approx$	$\approx$	$\approx$	$\approx$	$\approx$	$\approx$	$\approx$
**3D volumes**	**3D features fusion**	$\Uparrow$	$\approx$	$\approx$	$\approx$	$\approx$	$\approx$	$\approx$
**3D volumes**	**3D features fusion+2D biomarkers**	$\Uparrow$	$\approx$	$\approx$	$\approx$	$\approx$	$\approx$	$\approx$
**3D volumes**	**3D features fusion+3D biomarkers**	$\Uparrow$	$\approx$	$\approx$	$\approx$	$\approx$	$\approx$	$\approx$
**3D volumes**	**3D features fusion+2D biomarkers+3D biomarkers**	$\approx$	$\approx$	$\approx$	$\approx$	$\approx$	$\approx$	$\approx$
**3D fluid regions**	**3D concatenated**	$\approx$	$\approx$	$\Uparrow$	$\approx$	$\approx$	$\approx$	$\Uparrow$
**3D fluid regions**	**3D merged**	$\approx$	$\approx$	$\Uparrow$	$\Uparrow$	$\approx$	$\approx$	$\Uparrow$
**3D fluid regions**	**3D features fusion**	$\Uparrow$	$\Uparrow$	$\Uparrow$	$\approx$	$\Uparrow$	$\Uparrow$	$\Uparrow$
**3D fluid regions**	**3D features fusion+2D biomarkers**	$\Uparrow$	$\Uparrow$	$\Uparrow$	$\approx$	$\Uparrow$	$\Uparrow$	$\Uparrow$
**3D fluid regions**	**3D features fusion+3D biomarkers**	$\Uparrow$	$\Uparrow$	$\Uparrow$	$\approx$	$\Uparrow$	$\Uparrow$	$\Uparrow$
**3D fluid regions**	**3D features fusion+2D biomarkers+3D biomarkers**	$\Uparrow$	$\Uparrow$	$\Uparrow$	$\approx$	$\Uparrow$	$\Uparrow$	$\Uparrow$
**3D concatenated**	**3D merged**	$\approx$	$\approx$	$\approx$	$\approx$	$\approx$	$\approx$	$\approx$
**3D concatenated**	**3D features fusion**	$\Uparrow$	$\Uparrow$	$\approx$	$\approx$	$\approx$	$\Uparrow$	$\approx$
**3D concatenated**	**3D features fusion+2D biomarkers**	$\Uparrow$	$\Uparrow$	$\approx$	$\approx$	$\approx$	$\Uparrow$	$\approx$
**3D concatenated**	**3D features fusion+3D biomarkers**	$\Uparrow$	$\Uparrow$	$\approx$	$\approx$	$\approx$	$\Uparrow$	$\approx$
**3D concatenated**	**3D features fusion+2D biomarkers+3D biomarkers**	$\Uparrow$	$\Uparrow$	$\approx$	$\approx$	$\approx$	$\Uparrow$	$\approx$
**3D merged**	**3D features fusion**	$\Uparrow$	$\Uparrow$	$\Uparrow$	$\approx$	$\approx$	$\Uparrow$	$\approx$
**3D merged**	**3D features fusion+2D biomarkers**	$\Uparrow$	$\Uparrow$	$\Uparrow$	$\Uparrow$	$\approx$	$\Uparrow$	$\approx$
**3D merged**	**3D features fusion+3D biomarkers**	$\Uparrow$	$\Uparrow$	$\Uparrow$	$\Uparrow$	$\approx$	$\Uparrow$	$\approx$
**3D merged**	**3D features fusion+2D biomarkers+3D biomarkers**	$\Uparrow$	$\approx$	$\Uparrow$	$\Uparrow$	$\approx$	$\Uparrow$	$\approx$
**3D features fusion**	**3D features fusion+2D biomarkers**	$\approx$	$\approx$	$\approx$	$\approx$	$\approx$	$\approx$	$\approx$
**3D features fusion**	**3D features fusion+3D biomarkers**	$\approx$	$\approx$	$\approx$	$\approx$	$\approx$	$\approx$	$\approx$
**3D features fusion**	**3D features fusion+2D biomarkers+3D biomarkers**	$\approx$	$\approx$	$\approx$	$\approx$	$\approx$	$\approx$	$\approx$
**3D features fusion+2D biomarkers**	**3D features fusion+3D biomarkers**	$\approx$	$\approx$	$\approx$	$\approx$	$\approx$	$\approx$	$\approx$
**3D features fusion+2D biomarkers**	**3D features fusion+2D biomarkers+3D biomarkers**	$\approx$	$\approx$	$\approx$	$\approx$	$\approx$	$\approx$	$\approx$
**3D features fusion+3D biomarkers**	**3D features fusion+2D biomarkers+3D biomarkers**	$\approx$	$\approx$	$\approx$	$\approx$	$\approx$	$\approx$	$\approx$

### Comparison with Existing Literature

Given the absence of a publicly accessible labelled 3D CSCR dataset, a direct comparison with other methodologies is challenging. However, our proposed approach has shown encouraging results, aligning and in some instances surpassing, performance metrics set by state-of-the-art methodologies for similar tasks. These studies are discussed in Section 1.1.

With respect to PDT response prediction, the only study for comparison currently is Fernández-Vigo et al. [32]. As indicated in Table 9, our methodology has shown significant improvements over the Fernández-Vigo study in terms of accuracy, with an increase of 20.75%, 17.91%, and 36.76% for strategies 1, 2, and 3, respectively. Moreover, our method exhibits superior performance across all groups and strategies, as reflected by higher metrics. In addition, our method demonstrates lower standard deviation values, suggesting a potential for more stable and robust predictions. This improved performance can be primarily attributed to the efficient use of the 3D features of the image and the 3D fluid region generated by our method.

The primary contributions of this work are multifaceted, bridging DL techniques and the clinical field of retinal disorder diagnosis and management. Our efforts push the boundaries of current practice in automated OCT analysis, paving the way for improved patient outcomes and more impactful research studies. They are summarized as follows:

- **3D CSCR Fluid Segmentation:** This research presents a cutting-edge DL methodology that leverages the 3D context present in OCT scans. By comprehending the volumetric attributes, the model facilitates a more refined segmentation of fluid regions, thereby enhancing the granularity of the derived insights.
- **Prediction of the Response to PDT:** Our model transcends traditional segmentation boundaries by using pre-treatment OCT images to foresee PDT response in chronic CSCR patients. This forward-looking feature could catalyze a shift in patient management, paving the way for bespoke treatment plans and the optimal realization of patient outcomes.
- **Clinical and Research Utility:** The outcomes of this study bear implications that extend beyond academia, impacting real-world clinical practices. By decreasing the time and effort required for segmentation, offering standardization, and facilitating extensive research studies, our model could significantly bolster the quality of patient care and propel research in the CSCR domain.

## Conclusions

The development and application of a reliable, automated system for the segmentation of fluid regions in OCT scans are paramount to the diagnosis, treatment, and research progression of CSCR. This study encapsulates a comprehensive exploration of this domain by leveraging state-of-the-art advancements in DL and AI.

The system proposed herein, built upon a 3D end-to-end fully convolutional architecture, has manifested encouraging results in the accurate segmentation of fluid regions in OCT scans. Upon assessing various network configurations, it was revealed that the 3D nnU-Net with full resolution rendered the most superior performance. This underscores the importance of exploiting the 3D information inherent in the scans to enhance segmentation results. The proposed system’s capacity to automate the segmentation process has far-reaching implications, including significant reductions in analysis time and effort, as well as enabling a more efficient and consistent processing of large datasets.

**Table 9:** Comparison of our top-performing approach for PDT response prediction with existing literature

**Strategy**	**Input**	**Group**	**Precision**	**Recall**	**F1-score**	**Accuracy**
1	Fernández-Vigo et al. [32]	1	$0.57\pm 0.07$	$0.77\pm 0.22$	$0.63\pm 0.08$	$0.53\pm 0.03$
		2	$0.54\pm 0.19$	$0.37\pm 0.20$	$0.39\pm 0.12$	
		3	$0.32\pm 0.28$	$0.27\pm 0.25$	$0.29\pm 0.26$	
	Our proposal	1	$0.75\pm 0.04$	$0.66\pm 0.16$	$0.70\pm 0.11$	$0.64\pm 0.06$
		2	$0.63\pm 0.04$	$0.62\pm 0.08$	$0.62\pm 0.04$	
		3	$0.52\pm 0.12$	$0.64\pm 0.10$	$0.57\pm 0.11$	
2	Fernández-Vigo et al. [32]	1	$0.66\pm 0.07$	$0.63\pm 0.18$	$0.63\pm 0.10$	$0.67\pm 0.05$
		2 + 3	$0.71\pm 0.09$	$0.70\pm 0.15$	$0.69\pm 0.07$	
	Our proposal	1	$0.76\pm 0.11$	$0.80\pm 0.14$	$0.78\pm 0.10$	$0.79\pm 0.09$
		2 + 3	$0.83\pm 0.11$	$0.79\pm 0.09$	$0.80\pm 0.08$	
3	Fernández-Vigo et al. [32]	2	$0.74\pm 0.12$	$0.75\pm 0.16$	$0.73\pm 0.06$	$0.68\pm 0.07$
		3	$0.69\pm 0.15$	$0.60\pm 0.29$	$0.58\pm 0.21$	
	Our proposal	2	$0.93\pm 0.06$	$0.95\pm 0.06$	$0.94\pm 0.06$	$0.93\pm 0.06$
		3	$0.94\pm 0.08$	$0.90\pm 0.09$	$0.92\pm 0.07$	

Furthermore, our work extends to the prediction of treatment responses before the administration of PDT. This aspect is crucial as it opens the door to personalized medicine, enabling treatment plans to be tailored based on individual patient characteristics, thus optimizing effectiveness while minimizing adverse effects. An early prediction approach also contributes to the avoidance of unnecessary treatments, thereby saving valuable resources.

Our PDT response analysis yielded results that underlined the effectiveness of our proposed methodologies, significantly outperforming the established baselines. Particularly, our 3D feature fusion approach demonstrated substantial improvements in both accuracy and precision. The capability to accurately predict treatment responses allows clinicians to strategically optimize treatment plans and closely monitor patients’ progress, thus contributing to enhanced patient outcomes and quality of life.

Overall, this research delivers significant contributions to the field of ophthalmology, presenting an accurate and efficient system for fluid segmentation and treatment response prediction in CSCR patients. The system proposed offers multiple advantages over manual segmentation, notably increased speed, consistency, and accuracy. It harbors the potential to bring about a paradigm shift in the diagnosis and treatment of CSCR, ultimately leading to improved patient outcomes and aiding the furtherance of research in the field.

There are some limitations to this study though. While the usage of OCT technique has effectively revealed detailed aspects of retinal pathology, we acknowledge that expanding the range of imaging modalities could enrich our understanding. Including techniques such as fundus photography, OCT-A and fluorescein angiography in future studies would allow for a broader evaluation of disease mechanisms and enhance the comprehensiveness of our findings. Additionally, the three-month follow-up period utilized in our study to assess the PDT response effectively allowed us to evaluate its immediate effects. However, to more fully understand the long-term impacts and stability of these treatments, future research should consider extending this follow-up period. Such extensions would provide valuable insights into the long-term efficacy of treatments and potential recurrences.

Future works could look to integrate larger and more diverse datasets to amplify the system’s robustness and generalizability. Additionally, the incorporation of specific biomarkers and other relevant patient information could be explored to analyze their relation with the patient’s response to treatment.

## References

[1] M. Wang, I.C. Munch, P.W. Hasler, C. Prünte, M. Larsen, Central serous chorioretinopathy. Acta ophthalmologica **86**(2), 126–145 (2008). 10.1111/j.1600-0420.2007.00889.x 17662099 10.1111/j.1600-0420.2007.00889.x

[2] D.C. Tsai, S.J. Chen, C.C. Huang, P. Chou, C.M. Chung, P.H. Huang, S.J. Lin, J.W. Chen, T.J. Chen, H.B. Leu, et al., Epidemiology of idiopathic central serous chorioretinopathy in taiwan, 2001–2006: a population-based study. PloS one **8**(6), e66,858 (2013). 10.1371/journal.pone.0066858 10.1371/journal.pone.0066858 PMC3691239 23826160

[3] L.A. Yannuzzi, J.L. Shakin, Y.L. Fisher, M.A. Altomonte, Peripheral retinal detachments and retinal pigment epithelial atrophic tracts secondary to central serous pigment epitheliopathy. Ophthalmology **91**(12), 1554–1572 (1984). 10.1016/S0161-6420(84)34117-3 6084221 10.1016/s0161-6420(84)34117-3

[4] J.I. Lim, A.R. Glassman, L.P. Aiello, U. Chakravarthy, C.J. Flaxel, R.F. Spaide, M.S.C.C.S. Group, Research, E. Committee, W. Committee, et al., Collaborative retrospective macula society study of photodynamic therapy for chronic central serous chorioretinopathy. Ophthalmology **121**(5), 1073–1078 (2014). 10.1016/j.ophtha.2013.11.040 10.1016/j.ophtha.2013.11.040 24439758

[5] W.M. Chan, T.Y. Lai, R.Y. Lai, D.T. Liu, D.S. Lam, Half-dose verteporfin photodynamic therapy for acute central serous chorioretinopathy: one-year results of a randomized controlled trial. Ophthalmology **115**(10), 1756–1765 (2008). 10.1016/j.ophtha.2008.04.014 18538401 10.1016/j.ophtha.2008.04.014

[6] E.H. Van Dijk, S. Fauser, M.B. Breukink, R. Blanco-Garavito, J.M. Groenewoud, J.E. Keunen, P.J. Peters, G. Dijkman, E.H. Souied, R.E. MacLaren, et al., Half-dose photodynamic therapy versus high-density subthreshold micropulse laser treatment in patients with chronic central serous chorioretinopathy: the place trial. Ophthalmology **125**(10), 1547–1555 (2018). 10.1016/j.ophtha.2018.04.021 29776672 10.1016/j.ophtha.2018.04.021

[7] Y. Kon, T. Iida, I. Maruko, M. Saito, The optical coherence tomography–ophthalmoscope for examination of central serous chorioretinopathy with precipitates. Retina **28**(6), 864–869 (2008). 10.1097/IAE.0b013e3181669795 18536604 10.1097/IAE.0b013e3181669795

[8] S. Mrejen, R.F. Spaide, Optical coherence tomography: imaging of the choroid and beyond. Survey of ophthalmology **58**(5), 387–429 (2013). 10.1016/j.survophthal.2012.12.001 23916620 10.1016/j.survophthal.2012.12.001

[9] J. Ruiz-Medrano, L. Arias, J.M. Ruiz-Moreno, in *Central Serous Chorioretinopathy* (2019), pp. 115–128. 10.1016/B978-0-12-816800-4.00010-3

[10] O. Ronneberger, P. Fischer, T. Brox, in *Medical Image Computing and Computer-Assisted Intervention–MICCAI 2015: 18th International Conference, Munich, Germany, October 5-9, 2015, Proceedings, Part III 18* (Springer, 2015), pp. 234–241. 10.1007/978-3-319-24574-4_28

[11] A. Esteva, B. Kuprel, R.A. Novoa, J. Ko, S.M. Swetter, H.M. Blau, S. Thrun, Dermatologist-level classification of skin cancer with deep neural networks. nature **542**(7639), 115–118 (2017). 10.1038/nature21056 10.1038/nature21056 PMC8382232 28117445

[12] M. Chen, K. Jin, K. You, Y. Xu, Y. Wang, C.C. Yip, J. Wu, J. Ye, Automatic detection of leakage point in central serous chorioretinopathy of fundus fluorescein angiography based on time sequence deep learning. Graefe’s Archive for Clinical and Experimental Ophthalmology **259**, 2401–2411 (2021). 10.1007/s00417-021-05151-x 33846835 10.1007/s00417-021-05151-x

[13] F. Xu, S. Liu, Y. Xiang, Z. Lin, C. Li, L. Zhou, Y. Gong, L. Li, Z. Li, C. Guo, et al., Deep learning for detecting subretinal fluid and discerning macular status by fundus images in central serous chorioretinopathy. Frontiers in Bioengineering and Biotechnology **9**, 651,340 (2021). 10.3389/fbioe.2021.651340 10.3389/fbioe.2021.651340 PMC8604280 34805102

[14] T.K. Yoo, B.Y. Kim, H.K. Jeong, H.K. Kim, D. Yang, I.H. Ryu, Simple code implementation for deep learning–based segmentation to evaluate central serous chorioretinopathy in fundus photography. Translational Vision Science & Technology **11**(2), 22–22 (2022). 10.1167/tvst.11.2.22 10.1167/tvst.11.2.22 PMC8842634 35147661

[15] C.S. Lee, D.M. Baughman, A.Y. Lee, Deep learning is effective for classifying normal versus age-related macular degeneration oct images. Ophthalmology Retina **1**(4), 322–327 (2017). 10.1016/j.oret.2016.12.009 30693348 10.1016/j.oret.2016.12.009 PMC6347658

[16] J. De Moura, J. Novo, S. Penas, M. Ortega, J. Silva, A.M. Mendonça, Automatic characterization of the serous retinal detachment associated with the subretinal fluid presence in optical coherence tomography images. Procedia Computer Science **126**, 244–253 (2018). 10.1016/j.procs.2018.07.258

[17] P.L. Vidal, J. De Moura, J. Novo, M.G. Penedo, M. Ortega, Intraretinal fluid identification via enhanced maps using optical coherence tomography images. Biomedical optics express **9**(10), 4730–4754 (2018). 10.1364/BOE.9.004730 30319899 10.1364/BOE.9.004730 PMC6179401

[18] M. Gende, J. De Moura, J.I. Fernández-Vigo, J.M. Martínez-de-la Casa, J. García-Feijóo, J. Novo, M. Ortega, Robust multi-view approaches for retinal layer segmentation in glaucoma patients via transfer learning. Quantitative Imaging in Medicine and Surgery **13**(5), 2846 (2023). 10.21037/qims-22-959 10.21037/qims-22-959 PMC10167471 37179949

[19] G. Girish, B. Thakur, S.R. Chowdhury, A.R. Kothari, J. Rajan, Segmentation of intra-retinal cysts from optical coherence tomography images using a fully convolutional neural network model. IEEE journal of biomedical and health informatics **23**(1), 296–304 (2018). 10.1109/JBHI.2018.2810379 29994161 10.1109/JBHI.2018.2810379

[20] K. Gao, W. Kong, S. Niu, D. Li, Y. Chen, Automatic retinal layer segmentation in sd-oct images with csc guided by spatial characteristics. Multimedia Tools and Applications **79**, 4417–4428 (2020). 10.1007/s11042-019-7395-9

[21] T.N. Rao, G. Girish, A.R. Kothari, J. Rajan, in *2019 41st Annual International Conference of the IEEE Engineering in Medicine and Biology Society (EMBC)* (IEEE, 2019), pp. 978–981. 10.1109/EMBC.2019.8857105 10.1109/EMBC.2019.8857105 31946057

[22] J. De Moura, J. Novo, M. Ortega, N. Barreira, M.G. Penedo, in *2021 IEEE 34th International Symposium on Computer-Based Medical Systems (CBMS)* (IEEE, 2021), pp. 1–6. 10.1109/CBMS52027.2021.00008

[23] F.H. Lai, D.S. Ng, M. Bakthavatsalam, V.C. Chan, A.L. Young, F.O. Luk, C.W. Tsang, M.E. Brelén, A multicenter study on the long-term outcomes of half-dose photodynamic therapy in chronic central serous chorioretinopathy. American journal of ophthalmology **170**, 91–99 (2016). 10.1016/j.ajo.2016.07.026 27519561 10.1016/j.ajo.2016.07.026

[24] K. Fujita, Y. Imamura, K. Shinoda, C.S. Matsumoto, Y. Mizutani, K. Hashizume, A. Mizota, M. Yuzawa, One-year outcomes with half-dose verteporfin photodynamic therapy for chronic central serous chorioretinopathy. Ophthalmology **122**(3), 555–561 (2015). 10.1016/j.ophtha.2014.09.034 25444637 10.1016/j.ophtha.2014.09.034

[25] T.J. Van Rijssen, E.H. Van Dijk, S. Yzer, K. Ohno-Matsui, J.E. Keunen, R.O. Schlingemann, S. Sivaprasad, G. Querques, S.M. Downes, S. Fauser, et al., Central serous chorioretinopathy: towards an evidence-based treatment guideline. Progress in Retinal and Eye Research **73**, 100,770 (2019). 10.1016/j.preteyeres.2019.07.003 10.1016/j.preteyeres.2019.07.003 31319157

[26] R. Poplin, A.V. Varadarajan, K. Blumer, Y. Liu, M.V. McConnell, G.S. Corrado, L. Peng, D.R. Webster, Prediction of cardiovascular risk factors from retinal fundus photographs via deep learning. Nature biomedical engineering **2**(3), 158–164 (2018). 10.1038/s41551-018-0195-0 31015713 10.1038/s41551-018-0195-0

[27] V. Gulshan, L. Peng, M. Coram, M.C. Stumpe, D. Wu, A. Narayanaswamy, S. Venugopalan, K. Widner, T. Madams, J. Cuadros, et al., Development and validation of a deep learning algorithm for detection of diabetic retinopathy in retinal fundus photographs. jama **316**(22), 2402–2410 (2016). 10.1001/jama.2016.17216 10.1001/jama.2016.17216 27898976

[28] J. De Fauw, J.R. Ledsam, B. Romera-Paredes, S. Nikolov, N. Tomasev, S. Blackwell, H. Askham, X. Glorot, B. O’Donoghue, D. Visentin, et al., Clinically applicable deep learning for diagnosis and referral in retinal disease. Nature medicine **24**(9), 1342–1350 (2018). 10.1038/s41591-018-0107-6 30104768 10.1038/s41591-018-0107-6

[29] Y. Zhen, H. Chen, X. Zhang, X. Meng, J. Zhang, J. Pu, Assessment of central serous chorioretinopathy depicted on color fundus photographs using deep learning. Retina **40**(8), 1558–1564 (2020). 10.1097/IAE.0000000000002621 31283737 10.1097/IAE.0000000000002621

[30] W. Chan, D. Lam, T. Lai, B. Tam, D. Liu, C. Chan, Choroidal vascular remodelling in central serous chorioretinopathy after indocyanine green guided photodynamic therapy with verteporfin: a novel treatment at the primary disease level. British Journal of Ophthalmology **87**(12), 1453–1458 (2003). 10.1136/bjo.87.12.1453 14660450 10.1136/bjo.87.12.1453 PMC1920573

[31] F. Xu, C. Wan, L. Zhao, S. Liu, J. Hong, Y. Xiang, Q. You, L. Zhou, Z. Li, S. Gong, et al., Predicting post-therapeutic visual acuity and oct images in patients with central serous chorioretinopathy by artificial intelligence. Frontiers in Bioengineering and Biotechnology **9**, 649,221 (2021). 10.3389/fbioe.2021.649221 10.3389/fbioe.2021.649221 PMC8650495 34888298

[32] J.I. Fernández-Vigo, V.G. Calleja, J.J. De Moura Ramos, J. Novo-Bujan, B. Burgos-Blasco, L. López-Guajardo, J. Donate-López, M. Ortega-Hortas, Prediction of the response to photodynamic therapy in patients with chronic central serous chorioretinopathy based on optical coherence tomography using deep learning. Photodiagnosis and Photodynamic Therapy **40**, 103,107 (2022). 10.1016/j.pdpdt.2022.103107 10.1016/j.pdpdt.2022.103107 36070850

[33] J. Chhablani, F.B. Cohen, P. Aymard, T. Beydoun, E. Bousquet, A. Daruich-Matet, A. Matet, M. Zhao, C.M.G. Cheung, K.B. Freund, et al., Multimodal imaging-based central serous chorioretinopathy classification. Ophthalmology Retina **4**(11), 1043–1046 (2020). 10.1016/j.oret.2020.07.026 33131671 10.1016/j.oret.2020.07.026

[34] J.I. Fernández-Vigo, F.J. Moreno-Morillo, A. Valverde-Megías, B. Burgos-Blasco, L. López-Guajardo, J. Donate-López, Acute exudative maculopathy and bacillary layer detachment in patients with central serous chorioretinopathy after photodynamic therapy. Retina **42**(5), 859–866 (2022). 10.1097/IAE.0000000000003404 35019888 10.1097/IAE.0000000000003404

[35] F. Isensee, P.F. Jaeger, S.A. Kohl, J. Petersen, K.H. Maier-Hein, nnu-net: a self-configuring method for deep learning-based biomedical image segmentation. Nature methods **18**(2), 203–211 (2021). 10.1038/s41592-020-01008-z 33288961 10.1038/s41592-020-01008-z

[36] M. Brett, C.J. Markiewicz, M. Hanke, M.A. Côté, B. Cipollini, P. McCarthy, D. Jarecka, C.P. Cheng, Y.O. Halchenko, M. Cottaar, E. Larson, S. Ghosh, D. Wassermann, S. Gerhard, G.R. Lee, H.T. Wang, E. Kastman, J. Kaczmarzyk, R. Guidotti, freec84. nipy/nibabel: (4.0.0) (2022). 10.5281/zenodo.6658382

[37] MONAI Consortium. Monai: Medical open network for ai (2023). 10.5281/zenodo.8018287

[38] F. Pedregosa, G. Varoquaux, A. Gramfort, V. Michel, B. Thirion, O. Grisel, M. Blondel, P. Prettenhofer, R. Weiss, V. Dubourg, et al., Scikit-learn: Machine learning in python. the Journal of machine Learning research **12**, 2825–2830 (2011). 10.48550/arXiv.1201.0490

[39] S.A. Taghanaki, Y. Zheng, S.K. Zhou, B. Georgescu, P. Sharma, D. Xu, D. Comaniciu, G. Hamarneh, Combo loss: Handling input and output imbalance in multi-organ segmentation. Computerized Medical Imaging and Graphics **75**, 24–33 (2019). 10.1016/j.compmedimag.2019.04.005 31129477 10.1016/j.compmedimag.2019.04.005

[40] N. Heller, F. Isensee, K.H. Maier-Hein, X. Hou, C. Xie, F. Li, Y. Nan, G. Mu, Z. Lin, M. Han, et al., The state of the art in kidney and kidney tumor segmentation in contrast-enhanced ct imaging: Results of the kits19 challenge. Medical image analysis **67**, 101,821 (2021). 10.1016/j.media.2020.101821 10.1016/j.media.2020.101821 PMC7734203 33049579

[41] F. Quinton, B. Presles, S. Leclerc, G. Nodari, O. Lopez, O. Chevallier, J. Pellegrinelli, J.M. Vrigneaud, R. Popoff, F. Meriaudeau, et al., Navigating the nuances: comparative analysis and hyperparameter optimisation of neural architectures on contrast-enhanced mri for liver and liver tumour segmentation. Scientific Reports **14**(1), 3522 (2024). 10.1038/s41598-024-53528-9 38347017 10.1038/s41598-024-53528-9 PMC10861452

[42] J. Wang, L. Perez, et al., The effectiveness of data augmentation in image classification using deep learning. Convolutional Neural Networks Vis. Recognit **11**(2017), 1–8 (2017). 10.48550/arXiv.1712.04621

[43] G. Samagaio, J. De Moura, J. Novo, M. Ortega, Automatic segmentation of diffuse retinal thickening edemas using optical coherence tomography images. Procedia Computer Science **126**, 472–481 (2018). 10.1016/j.procs.2018.07.281

[44] G. Huang, Z. Liu, L. Van Der Maaten, K.Q. Weinberger, in *Proceedings of the IEEE conference on computer vision and pattern recognition* (2017), pp. 4700–4708. 10.48550/arXiv.1608.06993

[45] C. Cortes, V. Vapnik, Support-vector networks. Machine learning **20**, 273–297 (1995). 10.1007/BF00994018

[46] M.Y. Khachane, Organ-based medical image classification using support vector machine. International Journal of Synthetic Emotions (IJSE) **8**(1), 18–30 (2017). 10.4018/IJSE.2017010102

[47] C.S. Lo, C.M. Wang, Support vector machine for breast mr image classification. Computers & Mathematics with Applications **64**(5), 1153–1162 (2012). 10.1016/j.camwa.2012.03.033

[48] K. Sharma, J. Virmani, A decision support system for classification of normal and medical renal disease using ultrasound images: a decision support system for medical renal diseases. International Journal of Ambient Computing and Intelligence (IJACI) **8**(2), 52–69 (2017). 10.4018/IJACI.2017040104

[49] S. Ganesan, T. Subashini, K. Jayalakshmi, in *2014 International Conference on Communication and Signal Processing* (IEEE, 2014), pp. 1109–1112. 10.1109/ICCSP.2014.6950020

[50] D.P. Kingma, J. Ba, Adam: A method for stochastic optimization. arXiv preprint arXiv:1412.6980 (2014). 10.48550/arXiv.1412.6980

